# Current opinion: antiretrovirals during pregnancy and breastfeeding

**DOI:** 10.1097/COH.0000000000000884

**Published:** 2024-09-20

**Authors:** Laura Nijboer, Lena van der Wekken-Pas, Karoline Aebi-Popp, Elisabeth van Leeuwen, Angela Colbers

**Affiliations:** a Department of Pharmacy, Radboud Institute for Medical Innovations (RIMI), Radboud University Medical Center, Nijmegen, The Netherlands; b Department of Infectious Diseases, Bern University Hospital; c Department of Obstetrics and Gynecology, Lindenhofspital, Bern, Switzerland; d Department of Obstetrics, Amsterdam Reproduction and Development, Amsterdam University Medical Center, Amsterdam, The Netherlands

**Keywords:** antiretrovirals, breast-feeding/chest-feeding, HIV, pharmacokinetics, pregnancy

## Abstract

**Purpose of review:**

To review the most important literature from the past 2 years on the pharmacokinetics of antiretrovirals in pregnancy, placental transfer, and breastmilk.

**Recent findings:**

Concentrations of antiretrovirals frequently used in pregnancy and their placental transfer are described, together with infant exposure through breastmilk. Also, applications of ex-vivo and in-silico studies, such as placenta perfusion studies and PBPK models, are discussed.

**Summary:**

Great efforts were made in the past 2 years to accelerate the availability of data on antiretrovirals during pregnancy and lactation. Bictegravir showed decreased but still sufficient concentrations during pregnancy, leading to a label change by the FDA. In-silico and clinical studies on long-acting cabotegravir and rilpivirine generated information leading to cautious use of these formulations in pregnancy. Low infant exposure to antiretrovirals through breastmilk is expected for most compounds. Despite the impact of these studies, more incentives are needed for earlier implementation, for instance, during the developmental phase of drugs, to provide women antenatally with proper information on their drugs.

## INTRODUCTION

*Though the terms ‘women’ and ‘breastfeeding’ are used throughout this paper, it is important to recognize that some who experience pregnancy and breastfeed do not identify as women. This paper is meant to be inclusive of all who experience pregnancy and are breastfeeding, regardless of gender identity.*

In 2022, globally, 1.2 million women living with HIV were pregnant [1]. Treatment with antiretrovirals decreases the risk of vertical transmission of HIV during pregnancy, labor, and breastfeeding, also includes chest-feeding, due to viral suppression [1]. However, selecting an appropriate antiretroviral treatment is challenging as drug levels change during pregnancy, and standard doses may not be adequate [2–4]. Reduced drug levels may lead to virological failure, resistance development, and vertical transmission [5]. Pharmacodynamic and pharmacokinetic research can determine the effectiveness of antiretrovirals during pregnancy, and the effect of physiological changes on pharmacokinetic parameters, while determining the safety at the same time.

Most antiretrovirals are transferred over the placenta to the fetus. Antiretrovirals may protect the fetus from HIV infection but may also cause harm if the drug has teratogenic potential or accumulates in the fetus [5]. Knowledge about the placental transfer of antiretrovirals is crucial to give recommendations regarding treatment of pregnant women.

In many (low-resource, middle-resource, and high-resource) settings, breastfeeding is acknowledged as an option for mothers living with HIV who have an undetectable viral load throughout pregnancy [1,6]. The benefits of breastfeeding may outbalance the low risk of vertical HIV transmission. First, increased bonding with the infant, as breastfeeding is considered the most natural feeding for neonates in many cultures [1]. Second, infants have a lower risk for sudden infant death, allergies, diabetes, and obesity [1]. Third, mothers who breastfeed have a reduced risk of breast and ovarian cancers [1]. It is important to understand the transfer of antiretrovirals into breastmilk, and infant exposure to antiretrovirals during breastfeeding.

This review provides an overview of the most important literature on the pharmacokinetics of antiretrovirals in pregnancy and breastfeeding published during 2022–2023. 

**Box 1:**
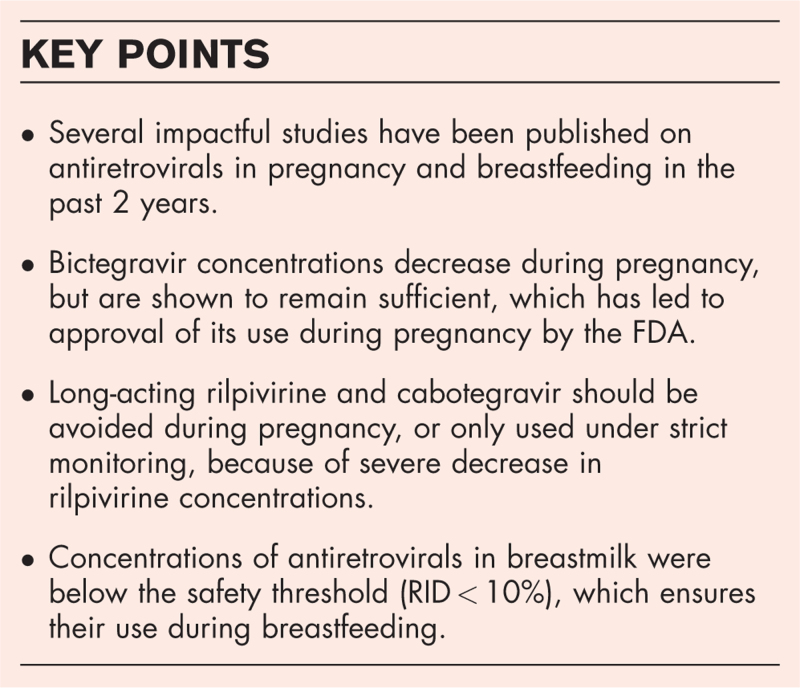
no caption available

## PREGNANCY PHARMACOKINETICS

**FIGURE 1:**
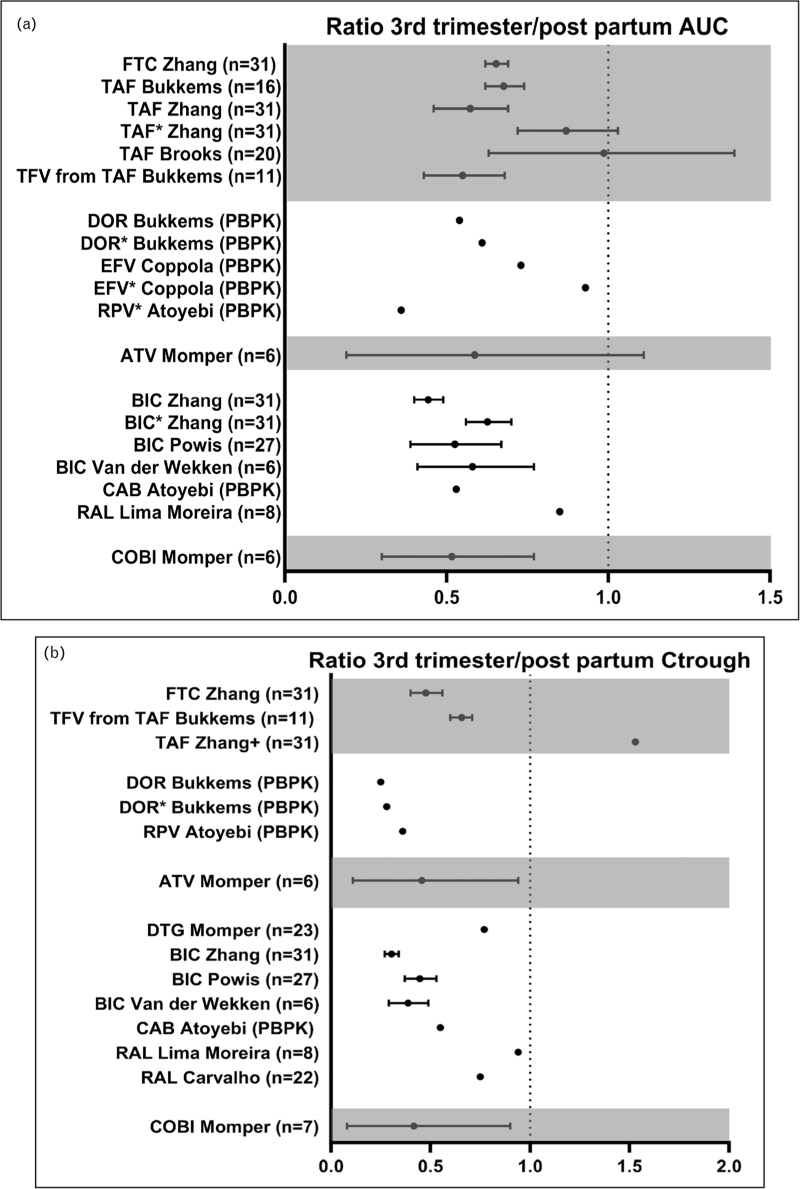
Ratio of area under the curve and *C*_trough_ in third trimester/postpartum. (∗) Unbound drug; (+) *C*_last_.

In the past 2 years, 15 studies have reported on antiretroviral pharmacokinetics during pregnancy. Four studies used physiologically based pharmacokinetic (PBPK) models and will be discussed later in the review. A summary of the results of the remaining 11 studies are shown in Fig. 1a and b and Supplementary Table 1.

## NUCLEOSIDE REVERSE TRANSCRIPTASE DRUGS

Nucleoside reverse transcriptase drugs (NRTIs) are part of all US Department of Health and Human Services (DHHS) and European AIDS Clinical Society (EACS)-preferred perinatal regimens [7,8]. Tenofovir alafenamide (TAF) is a frequently used NRTI in high-income settings and is a prodrug of tenofovir. Both compounds have lower concentrations in pregnancy in comparison to nonpregnant women [9^▪^,10^▪^,^11^^▪▪^]. However, the effect of pregnancy on tenofovir is less as compared with TAF. The decrease of TAF concentrations during pregnancy might be explained by decreased binding capacity of plasma proteins during pregnancy, as Zhang *et al.*[11^▪▪^] found a lower (16%) decrease in unbound TAF, than total TAF (37%) area under the curve (AUC). Also, TAF is a substrate of the transporters P-gp and BCRP, which are known to be induced during pregnancy and might affect its exposure. Interestingly, the magnitude of decrease in TAF concentrations differed between three studies [9^▪^,10^▪^,^11^^▪▪^], which is probably explained by differences in sample schedule. A more intensive sampling scheme ensures a more accurate capture of the absorption phase of the pharmacokinetic profile [10^▪^]. The results of the recently conducted studies have led to the inclusion of TAF as recommended NRTI in pregnancy without dose modification.

For another often used NRTI – emtricitabine (FTC) – the AUC was found to be decreased by 35% during the third trimester [11^▪▪^], which was in line with previously published reports [12,13]. The reduction of FTC and TAF in combination with bictegravir was not associated with virologic failure or vertical transmission [11^▪▪^,^12^,^13^], therefore, no dose adjustment is required for FTC and TAF during pregnancy [7,8].

## PROTEASE INHIBITORS

The preferred protease inhibitor during pregnancy is darunavir [7,8] in combination with ritonavir and NRTI backbone. A study with monotherapy darunavir/ritonavir twice daily 600/100 mg in pregnant women [14] showed that the total darunavir *C*_min_ remained stable during pregnancy, and was in line with *C*_min_ in pregnancy during triple therapy [14–16]. However, in nearly 10% of the cases, intensification (adding NRTIs) was needed to maintain viral suppression, therefore, darunavir/ritonavir monotherapy should not be recommended in pregnancy.

Protease inhibitors are metabolized by CYP3A4, and exposure to these drugs can be enhanced (boosted) by co-administration with cobicistat or ritonavir (CYP3A4 inhibitors). Previous studies have shown that boosting with cobicistat led to insufficient exposure to protease inhibitors during pregnancy [17]. Since 2019, cobicistat should not be used during pregnancy because of its reduced boosting effect [18]. Momper *et al.*[18] confirmed this for atazanavir/cobicistat. Reduced cobicistat AUC during the third trimester led to a more pronounced and clinically relevant decrease in atazanavir AUC (54%) compared with when atazanavir was combined with ritonavir (30–44%) and should not be recommended [18–20].

## INTEGRASE STRAND TRANSFER INHIBITORS

Bictegravir is a second-generation integrase strand transfer inhibitor (INSTI), for which little information was known regarding its use during pregnancy. It is metabolized by CYP3A4 and UGT1A1 enzymes. The activity of these enzymes is known to increase during pregnancy, and consequentially bictegravir concentrations are expected to be reduced. Three studies confirmed this reduction: the AUC was decreased by approximately 50% and *C*_trough_ by 60–70% in the third trimester compared with postpartum [11^▪▪^,^21^,^22^]. In all studies, bictegravir trough levels were above PA-IC95 of 0.162 mg/l, and no virological failure or vertical transmission occurred. The study of Zhang *et al.* led to advice in the label: continuation of treatment with bictegravir during pregnancy is possible if the potential benefits outweigh the potential risk to the fetus [23].

The total concentration of dolutegravir, another INSTI, is known to be decreased during pregnancy. Due to dolutegravir's high protein binding (>99%) and lower plasma protein concentrations during pregnancy, unbound dolutegravir during pregnancy may reflect clinical efficacy better than total concentration [24]. Momper *et al.*[24] studied unbound dolutegravir and found a decrease of 23% in *C*_trough_ in the third trimester versus postpartum and concluded that the standard dolutegravir-dosing advice is sufficient in pregnancy.

A recent study with raltegravir twice-daily 400 mg reported that raltegravir is metabolized to raltegravir-glucuronide mainly by UGT1A1 [25^▪^] and is a substrate of P-gp and BCRP. Previous studies showed that raltegravir pharmacokinetics are highly variable in pregnant and nonpregnant populations. Moreira *et al.*[25^▪^] reported approximately two-fold higher raltegravir-glucuronide metabolite formation clearance and elimination clearance in the third trimester versus postpartum, indicating UGT1A1 induction during the third trimester of pregnancy. The unbound raltegravir fraction did not seem to be affected during pregnancy [25^▪^]. Carvalho *et al.*[26] suggested to increase the minimal raltegravir *C*_trough_ to at least 0.04 mg/l in pregnancy (and not the standard 0.02 mg/l), as a predictor for virologic response in pregnancy. This suggestion is based on very few patients and there is no rationale given why the *C*_trough_ target in pregnancy should be different from the nonpregnant situation. Although standard dose of twice-daily raltegravir remains an option in pregnancy, increasingly, guidelines recommend dolutegravir as the preferred INSTI because of dosing once-daily and a higher genetic barrier to resistance.

## LONG-ACTING ANTIRETROVIRAL DRUGS

The first pharmacokinetic data on cabotegravir/rilpivirine long-acting injectables in pregnancy was reported by Patel *et al.*[27^▪^]. Women who became pregnant during clinical trials had to discontinue the injections, but for seven women, the pharmacokinetic tail of both compounds was recorded. The concentrations in the washout period during pregnancy were similar to those in nonpregnant women. For three out of seven women, cabotegravir plasma concentrations remained above the protein-adjusted IC90 (PA-IC90, 0.166 mg/l) for 9 months, and for 6 out of 7 women, rilpivirine plasma concentrations remained above the PA-IC90 (12 ng/ml) [27^▪^]. A case report by van der Wekken *et al.*[28^▪^] describes the concentrations throughout pregnancy and shows similar results for cabotegravir. However, rilpivirine concentrations were 74% lower in third trimester compared with 17 weeks postpartum. These data in combination with simulation done by Atoyebi *et al.*[29^▪▪^] might indicate that current long-acting formulations of cabotegravir/rilpivirine are not suitable during pregnancy or should at least be administered under close monitoring of viral load and plasma concentrations.

## PLACENTAL TRANSFER OF ANTIRETROVIRALS

To consider whether it is safe to use an antiretroviral during pregnancy, it is important to establish placental transfer. It can help to estimate the risk of toxicity, but may also give an impression of protection against vertical transmission of HIV if therapeutic levels are reached within the fetal circulation.

Placental transfer can be assessed clinically by obtaining maternal blood and cord blood at the time of delivery. Cord blood/maternal ratios (CB/MP ratio) of the antiretroviral concentrations have been regarded as a good surrogate for fetal exposure during third trimester [5]. Ideally, washout infant concentrations are also measured, because infant's metabolic capacity is often immature. Eke *et al.*[5] propose gathering at least two and a maximum of four samples up to eight times the (adult) half-life of the drug in infants during the washout phase. Ex-vivo (placenta perfusion studies) and in-silico (PBPK modelling) can also be used to estimate placental transfer (see paragraph on PBPK modelling).

**FIGURE 2:**
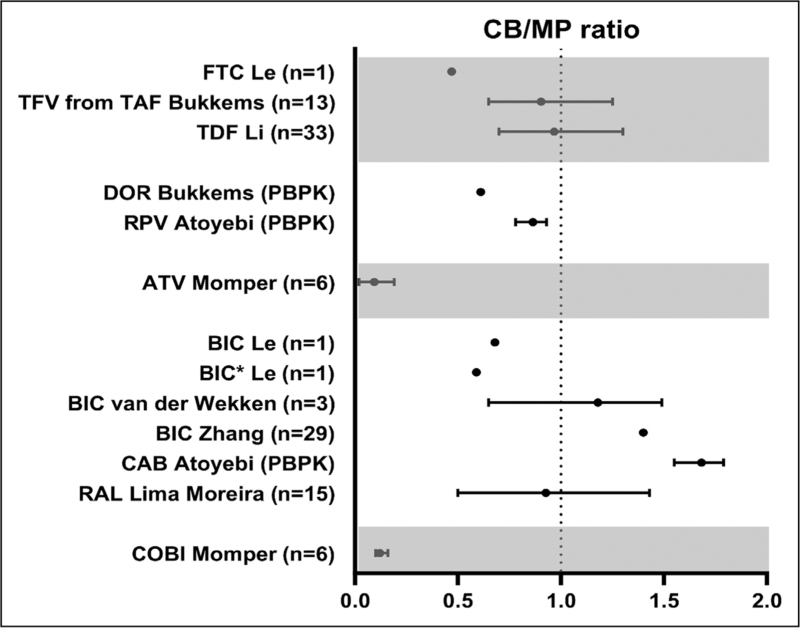
Cord blood/maternal plasma ratio. (∗) Unbound drug.

In the recent 2 years, several studies have reported on placental transfer of antiretrovirals. Results from these studies are summarized in Fig. 2.

Most studies reported on tenofovir and TAF. TAF concentrations were below the lower level of quantification in cord blood and almost all maternal blood samples [9^▪^,10^▪^,^11^^▪▪^,^30^]. Only for two paired samples, the CB/MP ratio could be calculated, which were 0.09 and 1.12. For tenofovir after ingestion of TAF, the reported CB/MP ratio of 0.81 (0.65–1.25) [10^▪^] was similar to the CB/MP ratio after tenofovir disoproxil (TDF) (between 0.26 and 1.95 [12,31,32]). Bictegravir CB/MP ratio was reported in a case report [30], and two prospective studies [11^▪▪^,^21^] and ranged from 0.65 to 1.49. With the use of a PBPK model, ratios for cabotegravir and rilpivirine after long-acting injectables were estimated to be 1.71 (1.55–1.79) and 0.88 (0.78–0.93), respectively [29^▪▪^]. CAB ratio is in line with other INSTIs, with a simulated ratio for CAB in line with the dolutegravir CB/MP ratio of 1.25 (1.07–1.40) [33]. Doravirine ratios were established to be 0.61 with a placental perfusion study and significant fetal exposure (concentrations >0.23 mg/l until after 8 h of maternal drug intake) was estimated with the subsequent developed PBPK model [34^▪^].

For the older drugs, reported CB/MP ratios were in concordance with previously reported ratios [12,17,35,36], except for atazanavir from which only data was available in case of boosting with ritonavir. Lower CB/MP ratios were found when boosted with cobicistat 0.07 (0.02–0.19) compared with 0.19 (0.10–0.32) [37] and 0.13 (0.10–0.16) [38] when boosted with ritonavir.

Washout infant concentrations were published for several drugs. For TAF and atazanavir, all levels were below detection limit [9^▪^,^11^^▪▪^,^18^]. Bictegravir could be measured in 10 samples and showed a half-life of 43.1 (38.4–57.6 h), which is almost 2.5 times the adult half-life [11^▪▪^], which is in line with dolutegravir [33]. This means that the neonate is exposed to bictegravir for some days after birth, which could help to prevent vertical transmission. If in the future, bictegravir was considered for infant postnatal prophylaxis, dosing should be determined by population and/or PBPK modeling.

## ANTIRETROVIRALS IN BREASTMILK

**FIGURE 3:**
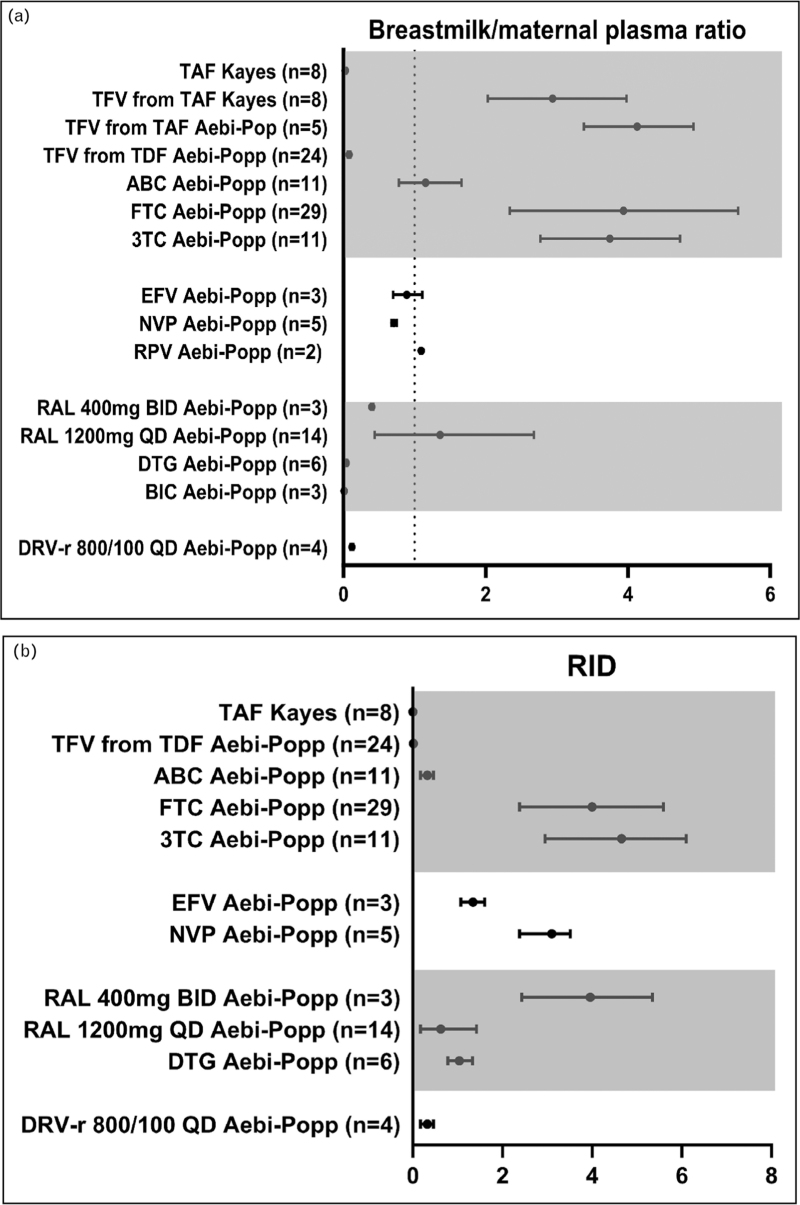
Breastmilk/maternal plasma ratio and relative infant dose.

In the past 2 years, three clinical studies reported transfer of antiretroviral drugs in breastmilk. Two were conducted in women with a chronic hepatitis B infection and described tenofovir after ingestion of either TAF or TDF [39^▪^,^40^], and one study in women living with HIV using several antiretrovirals [41^▪^]. Results are summarized in Figs. 3a and b and Table 1.

**Table 1:** Antiretrovirals in breastmilk

Drug	Time between drug intake and breastmilk sampling	BM/MP ratio	DID	RID	Absolute infant exposure	Reference
TAF (25 mg)	Maternal blood and breast milk sampling were taken at the following time points: predose. 0.5 h postdose. 1–1.5 h postdose. 2.5–3.5 h postdose. 5–6 h postdose. 8 h postdose and 24 h postdose	Not reported, but if median AUC and IQR are divided; follow ratios appear: 0.002 (0.003–0.02)	18.75 ng/kg/day	Median maternal weight was 64.9 kg and TAF dose 25 mg/day; therefore, the RID of TAF was calculated to be 0.005% of maternal dose of the drug		Kayes *et al.*[39^▪^]
TFV		2,91			TFV was detectable in 3/7 infant urine samples (rest BLQ); 12, 24 and 25 ng/ml	Kayes *et al.*[39^▪^]
EFV	Median and IQR 18.5 (18.0–18.8)	0.86 (0.70–1.11)	Median (IQR) mg/kg 0.41 (0.32–0.48)	1.35 (1.07–1.61) %	267 (17) ng/ml (time interval from maternal drug intake)	Aebi-Popp *et al.*[41^▪^]
NVP	14.5 (13.6–18.1)	0.70 (0.68–0.75)	0.41 (0.29–0.42)	3.40 (2.38–3.51)	960 (12); 227 (21)	Aebi-Popp *et al.*[41^▪^]
RPV	15.5 (15.3–15.8)	1.08 (1.1–1.1)	0.02 (0.02–0.02)	NA	4 (16)	Aebi-Popp *et al.*[41^▪^]
BIC	8.5 (5.5–11.5)	0.01 (0.01–0.01)	0.01 (0.01–0.01)	NA	103 (19)	Aebi-Popp *et al.*[41^▪^]
DTG	15 (13.0–19.7)	0.04 (0.03–0.05)	0.02 (0.01–0.02)	1.00 (0.78–1.33)	279 (15); 100 (13)	Aebi-Popp *et al.*[41^▪^]
RAL 400 mg b.i.d.	3.6 (3.1–5.7)	0.39 (0.39–0.42)	0.25 (0.15–0.32)	4.09 (2.43–5.35)	21 (2)	Aebi-Popp *et al.*[41^▪^]
RAL 1200 q.d.	13.7 (10.4–15.9)	0.96 (0.44–2.68)	0.02 (0.01–0.08)	0.28 (0.17–1.42)	0 (11); 0 (16); 0 (15); 14 (19); 0 (18); 0 (15)	Aebi-Popp *et al.*[41^▪^]
DRV 800/100 q.d.	15.4 (11.6–16.5)	0.12 (0.10–0.14)	0.05 (0.04–0.05)	0.34 (0.17–0.46)	0	Aebi-Popp *et al.*[41^▪^]
ABC	12.8 (10.3–13.6)	1.03 (0.78–1.66)	0.05 (0.04–0.05)	0.34 (0.17–0.46)	1 (11); 1 (18); 9 (13); 49 (12)	Aebi-Popp *et al.*[41^▪^]
FTC	15.5 (13.1–17.5)	3.92 (2.34–5.55)	0.12 (0.07–0.17)	4.02 (2.38–5.59)	44 (2); 21 (15); 5 (21); 14 (16); 18 (15); 27 (19); 0 (19); 26 (20); 0 (15); 49 (16); 24 (16)	Aebi-Popp *et al.*[41^▪^]
3TC	12.8 (10.3–13.6)	3.74 (2.77–4.73)	0.20 (0.12–0.24)	4.92 (2.95–6.10)	15 (11); 4 (18); 20 (13); 91 (12)	Aebi-Popp *et al.*[41^▪^]
TFV after TAF	8.5 (5.0–15.0)	4.09 (3.38–4.92)	0.007 (0.006–0.008)	NA	0	Aebi-Popp *et al.*[41^▪^]
TFV after TDF	16 (14.3–17.9)	0.08 (0.06–0.10)	0.001 (0.001–0.002)	0.01 (0.01–0.02)	0	Aebi-Popp *et al.*[41^▪^]

3TC, lamivudine; ABC, abacavir; BIC, bictegravir; b.i.d., twice daily; BM/MP ratio, breastmilk/maternal plasma ratio; DID, daily infant dose; DRV, darunavir; DTG, dolutegravir; EFV, efavirenz; FTC, emtricitabine; IQR, interquartile range; NA, not applicable; NVP, nevirapine; q.d., once daily; RAL, raltegravir; RID, relative infant dose; RPV, rilpivirine; TAF, tenofovir alafenamide; TDF, tenofovir disoproxil fumarate; TFV, tenofovir.

## NUCLEOSIDE REVERSE TRANSCRIPTASE DRUGS

In accordance with previous reports [42], breastmilk to maternal plasma ratios (MPRs) for most NRTIs were greater than 1, indicating transfer of these drugs into breastmilk. However, daily infant dose (DID) and relative infant dose (RID, weight-adjusted dose the infant ingests daily in relation to daily weight-adjusted maternal dose) for these drugs were far below safety threshold of 10%. Interestingly, the median concentrations for tenofovir after ingestion of TAF or TDF in breastmilk differed. The median tenofovir concentrations from TAF were 33.3 [interquartile range (IQR) 18.9–42.3] ng/ml [39^▪^] and 45 (37–54) ng/ml [41^▪^], while median concentration of tenofovir from TDF was 5 (4–10) ng/ml in one study [41^▪^] and *C*_24_ in another was 4.0 (2.7–6.1) ng/ml [40]. This difference may be caused by the greater lipophilicity of TAF compared with TDF, which allows easier transfer across mammarian membranes and diffusion into breastmilk. TAF is also a substrate of the transporters BCRP and P-gp, which are upregulated at mammarian epithelium during lactation, resulting in active transport into breastmilk. Also, TDF is rapidly converted to tenofovir in plasma, whereas TAF is converted to tenofovir only in peripheral blood mononuclear cells and hepatocytes resulting in lower tenofovir plasma concentrations, but a longer plasma half-life and therefore has a longer period of time to diffuse into breastmilk in comparison to TDF or tenofovir from TDF.

Although TAF dosing in pregnant women leads to higher concentrations of tenofovir in breastmilk in comparison to TDF, the absolute exposure of the infant to tenofovir is negligible. In four out of seven infants, the urine tenofovir concentrations were not detectable, the remaining urine concentrations were 300 times lower than previously measured in an adult population using TDF [39^▪^,^43^]. The low infant exposure to tenofovir is probably because of poor bioavailability, as tenofovir is a dianion at physiological pH, which reduces membrane permeability and hence absorption through the infant gut.

## NONNUCLEOSIDE REVERSE TRANSCRIPTASE INHIBITORS AND PROTEASE INHIBITORS

For the NNRTIs, a higher MPR for rilpivirine was reported than for nevirapine and efavirenz, however, for all NNRTIs, the DID and RID were low [3], which was also the case for the protease inhibitor darunavir (ritonavir-boosted). Unfortunately, the sample size for these drugs was low. Especially for efavirenz, only three samples were obtained from one woman, hampering interpretation because of lack of data on intersubject variability although, reassuringly, the findings were in line with previously published reports [42]. This also holds true for the protease inhibitors; only limited data on ritonavir-boosted darunavir (four samples from two women) were reported [3].

## INTEGRASE STRAND TRANSFER INHIBITORS

Integrase inhibitors showed variable breastmilk transfer [3]. Bictegravir and dolutegravir had low MPR, whereas MPR for raltegravir varied between used dosing regimens. The DID and RID for all INSTIs were low. Notably, the infant concentrations of dolutegravir and bictegravir were similar to, or higher than breastmilk concentrations, but concentrations remained 10–20-fold lower than trough concentrations reported for adults. This phenomenon is best explained by the ontogeny of CYP3A4 and UGT1A1, the enzymes metabolizing bictegravir and dolutegravir. CYP3A4 activity is low direct postnatally and increases after birth, reaching 50% of adult levels at 6–12 months of age [44]. Adult levels of UGT1A1 activity are reached within the first year of life [45]. Even though unmatured metabolization capacity in the exposed infants may lead to accumulation of integrase inhibitors through breastmilk, exposure is still low compared with adult concentrations.

So far, no RID above the safety threshold of 10% have been reported, which is reassuring. However, another concern is the occurrence of vertical transmission in a context of subtherapeutic concentrations. Usually, antiretrovirals with different half-lives and MPRs are prescribed together, and if for some of these drugs, subtherapeutic concentrations are reached in breastmilk, this might promote development of resistant strains of the virus in case of vertical transmission [46,47]. The DID for most drugs are already low, if also variable oral bioavailability is taken into account, most drugs will not reach therapeutic levels in the infant circulation.

## PHYSIOLOGICALLY BASED PHARMACOKINETIC MODELLING IN PREGNANCY AND LACTATION

As pregnant and lactating women are still denied access to participation in clinical studies, a time lag exists between approval of a drug and the moment pharmacokinetic data in pregnancy and lactation becomes available [48]. An approach to overcome this gap is PBPK modelling in which physicochemical properties of a drug are combined with physiological data of a specific population to predict pharmacokinetics.

Bukkems *et al.*[34^▪^] examined placental transfer of doravirine in a placenta perfusion study and used these data to parameterize a PBPK model to predict fetal and maternal exposure. Their experiments showed transfer of doravirine over placental tissue (fetal–maternal ratio 0.82). The model predicted a 55% decrease in maternal AUC_0–24_ and 84% in *C*_trough_ at 40 weeks gestation, compared with nonpregnant state. All *C*_trough_ levels during pregnancy were below the threshold of 0.23 mg/l, whereas simulations with a 100 mg twice-daily schedule showed an adequate *C*_trough_ throughout pregnancy. So, in anticipation of clinical data, these models predicted fetal exposure and that doravirine 100 mg once-daily probably leads to inadequate exposure during pregnancy.

Clinical data on cabotegravir and rilpivirine as long-acting injectables (LAI) in pregnancy are scarce. Cabotegravir/rilpivirine LAI are registered for monthly and bimonthly dosing and, both regimens have been shown to be equally effective in nonpregnant individuals [49,50], but according to the PBPK model of Atoyebi *et al.*[29^▪▪^], might not be suitable in pregnancy. The study showed slight decreases in cabotegravir exposure, but *C*_trough_ remained above the suggested target of 4xPA-IC90 (0.664 mg/l) in third trimester in all women on monthly dosing and 99.5% on a bimonthly dosing. For rilpivirine, however, exposures decreased by almost 70% and predicted rilpivirine *C*_trough_ was lower than the target of 50 ng/ml in approximately 50% using monthly injections and over 95% using a bimonthly schedule. This is an important warning for clinicians. In case a woman gets pregnant on this regimen and switching is not possible or desirable, close monitoring of viral load and drug concentrations, and shortening of the dosing interval should be considered.

Another application for PBPK models is to predict exposure in pregnancy and lactation in understudied populations for drugs, which are known to show variability because pf genotypic differences. Efavirenz for instance, is mainly metabolized by CYP2B6, of which multiple genotypes are associated with decreased or increased activity of these enzymes, leading to underexposure or toxicity. Pan and Rowland Yeo [51] used clinical data and a PBPK model to predict breastmilk concentrations for several genotypes and DID and subsequent infant exposures accounting for different infant genotypes. And even though differences per genotype were reported in this study, these were not clinically relevant and do not support genotyping before prescribing efavirenz in a lactating population. This interesting application of PBPK modeling could also be used for dolutegravir, as UGT1A1 polymorphism may influence exposure in pregnancy/lactation.

Peng *et al.*[52] used a PBPK model to predict fetal exposure to drugs that are known substrates of efflux pumps abundantly present in the placenta. As the abundance of these enzymes are time-dependent and in-vivo research can only provide data on placental transfer at the moment of delivery, a lack of data exists on transfer at earlier stages of pregnancy. With their model, they accurately predicted the unbound fraction transferred from fetus to mother (which resembles placental transfer) of nelfinavir and efavirenz at the second and third trimester and at delivery. The authors suggest their findings might support dose adjustment to better suit maternal and fetal benefits and risks, also for other drugs that are known transporter substrates. However, the use of nelfinavir and efavirenz is decreasing and on basis of clinical trials, dose adjustment is not recommended [7,8].

Lastly, PBPK models can be used to predict changes in total and unbound concentrations of drugs during/in pregnancy. In pregnancy, plasma protein binding and blood partitioning of drugs are changed because of changes in blood volume, hemodilution, and reduced concentration of the main drug-binding protein albumin and alpha-1-acid glycoprotein. Coppola *et al.*[53] used a PBPK model to predict the efavirenz unbound fraction (biologically active) in pregnancy. They predicted that although total concentrations of efavirenz decrease by 27%, which was in accordance with clinical data published previously, the unbound concentration only decreased by 7%.

PBPK models can attribute in gaining knowledge on pharmacokinetics in pregnancy and lactation, for example, for drugs where clinical data is pending, or to predict exposure in face of pharmacogenetic influences or to predict changes in total and unbound concentrations, which are to be expected during pregnancy.

## CONCLUSION

The incentives to accelerate pragmatic pharmacokinetic and PBPK modelling studies in pregnancy have the potential for considerable clinical impact as demonstrated by the recent change in the Food and Drug Administration (FDA) label supporting continuing bictegravir and the warning on the use of long-acting rilpivirine in pregnancy. In the past 2 years, more insight has been gained on breastmilk pharmacokinetics of antiretrovirals, which enables empowerment of women's desire to breastfeed. Incentives to implement similar studies during the developmental phase, before or shortly after drug approval, would further help to achieve the goal of providing women living with HIV preconceptionally with proper knowledge of their medication during the perinatal phase.

## Acknowledgements

*None.*

### Financial support and sponsorship

*None.*

### Conflicts of interest

*A.C. received research grants from ViiV Healthcare, Gilead, Merck, all paid to the institution. K.A.P. s institution received grants from Gilead and ViiV healthcare. L.N., L.v.d.W., and E.v.L. have no conflict of interest to report.*

## Supplementary Material

Supplemental Digital Content

## REFERENCES AND RECOMMENDED READING

Papers of particular interest, published within the annual period of review, have been highlighted as:

▪ of special interest
▪▪ of outstanding interest
