# Supplementary material for: Current opinion: antiretrovirals during pregnancy and breastfeeding
Source: Curr Opin HIV AIDS. 2024 Sep 20;19(6):305–15. doi: 10.1097/COH.0000000000000884 (PMC11451929; doi:10.1097/COH.0000000000000884)
Supplement: Supplemental Digital Content [file cohiv-19-305-s001.pdf]

Supplementary information for:

Current Opinion: antiretrovirals during pregnancy and breastfeeding

Table 1. Pregnancy effect on maternal pharmacokinetics

Drug (group)	Reference	Design	Population	AUC			C_{trough}			GMR				Comments
				Second trimester	Third trimester	Postpartum	Second trimester	Third trimester	Postpartum	AUC<br>2T/PP	 3T/PP	C_{trough}<br>2T/PP	 3T/PP	
**NRTIs**														
Emtricitabine														
	Zhang *et al.* [11••]	Open-label, single-arm, prospective, phase 1b study	T2:<br>n = 21<br>T3:<br>n = 30<br>PP:<br>n = 31	10.3 (20.0)	10.4 (20.3)	16.3 (24.7)	0.0598 (103.9)	0.0514 (27.2)	0.152 (178.5)	0.64 (0.61-0.68)	0.65 (0.62-0.69)	0.43 (0.37-0.50)	0.47 (0.40-0.56)	$AUC_{tau(unbound)}$: (h*mg/L) mean (%CV)<br>C_{trough}: mg/L<br>GMR (90% CI)<br><br>Only samples PP week 6 included.
Tenofovir Alafenamide														
	Zhang *et al.* [11••]	Open-label, single-arm, prospective phase 1b study	T2:<br>n = 21<br>T3:<br>n = 30<br>PP:<br>n = 31	Total:<br>0.236 (45.6)<br><br>Unbound:<br>0.015 (28.2)	Total:<br>0.212 (45.0)<br><br>Unbound:<br>0.016 (28.4)	Total:<br>0.374 (41.0)<br><br>Unbound:<br>0.018 (33.8)	C_{last}:<br>0.00449 (114.2)	C_{last}:<br>0.00480 (84.4)	C_{last}:<br>0.00313 (59.1)	Total:<br>0.63 (0.51-0.77)<br><br>Unbound:<br>0.84 (0.73-0.96)	Total:<br>0.57 (0.46-0.69)<br><br>Unbound:<br>0.86 (0.72-1.03)	C_{last}: 1.43	C_{last}: 1.53	$AUC_{tau(unbound)}$: (h*mg/L) mean (%CV)<br>C_{last}: mg/L<br>GMR (90% CI)<br><br>Only samples PP week 6 included.
	Brooks *et al.* [9•]	Prospective, opportunistic, open-label, multicenter, multi-arm phase IV study	T2:<br>n = 8<br>T3:<br>n = 20	181.1 (133.0-549.5)	257.2 (173.1-445.0)	283.3 (161.0-506.6)				0.62 (0.29-1.34)	0.94 (0.63-1.39)			AUC: ng*h/mL<br>C: ng/mL

Drug (group)	Reference	Design	Population	AUC			C_{trough}			GMR				Comments
				Second trimester	Third trimester	Postpartum	Second trimester	Third trimester	Postpartum	AUC		C_{trough}		
										2T/PP	3T/PP	2T/PP	3T/PP	
TAF														
	Bukkems *et al.* [10•]	Non-randomized, open-label, multicenter, phase IV study	T3: n = 17 PP: n = 12		101 (44)	217 (57)					0.54 (0.43-0.68)			LLOQ = 1 ng/mL 2 samples of T3 below LLOQ.<br><br>AUC: ng∗h/mL (CV%) C: ng/mL (CV%) GMR (90% CI)
Tenofovir after Tenofovir Alafenamide														
	Bukkems *et al.* [10•]	Non-randomized, open-label, multicenter, phase IV study	T3: n = 16 PP: n = 11		232 (30)	348.51 (33.3)		7 (36)	12 (38)		0.67 (0.62-0.74)		0.66 (0.60-0.71)	AUC: ng∗h/mL (CV%) C: ng/mL (CV%)
**NNRTIs**														
Doravirine														
	Bukkems *et al.* [34•]	Ex-vivo; PBPK model	n/a	Total: 10.7<br><br>Unbound: 3.16	Total: 9.33<br><br>Unbound: 2.82	Total: 17.32<br><br>Unbound: 4.66	Total: 0.10<br><br>Unbound: 0.03	Total: 0.07<br><br>Unbound: 0.02	Total: 0.28<br><br>Unbound: 0.08	Total: 0.62<br><br>Unbound: 0.68	Total, 0.54<br><br>Unbound, 0.61	Total: 0.35<br><br>Unbound: 0.39	Total: 0.25<br><br>Unbound: 0.28	AUC: mg*h/L C: mg/L<br><br>Only samples of T3 in week 32 included.
Efavirenz														
	Coppola *et al.* [53]	In silico; PBPK model	n/a	Total: 49.4<br><br>Unbound: 1.71	Total: 44.1<br><br>Unbound: 1.69	Total: 60.2<br><br>Unbound: 1.81				Total: 0.82<br><br>Unbound: 0.94	Total: 0.73<br><br>Unbound: 0.93			AUC: mg*h/L C: mg/L<br><br>Only plasma samples included.

Drug (group)	Reference	Design	Population	AUC			C_{trough}			GMR				Comments
				Second trimester	Third trimester	Postpartum	Second trimester	Third trimester	Postpartum	AUC		C_{trough}		
										2T/PP	3T/PP	2T/PP	3T/PP	
Rilpivirine														
	Atoyebi *et al.* [29••]	In silico; full-body adult PBPK model	n/a	Dose 1: 37130 (0.003)<br>Dose 2: 66795 (0.002)	Dose 1: 37648 (0.003)<br>Dose 2: 66038 (0.002)	Dose 1: 105703 (0.001)<br>Dose 2: 184491 (0.001)	Dose 1: 48 (2.25)<br>Dose 2: 39 (2.85)	Dose 1: 51 (2.22)<br>Dose 2: 40 (2.83)	Dose 1: 142 (0.82)<br>Dose 2: 110 (1.07)	Dose 1: 0.35<br>Dose 2: 0.36	Dose 1: 0.36<br>Dose 2: 0.36	Dose 1: 0.34<br>Dose 2: 0.35	Dose 1: 0.36<br>Dose 2: 0.36	AUC: ng*h/mL (CV%)<br>C: ng/mL (CV%)<br>Dose 1: 900mg then 600mg monthly<br>Dose 2: 900mg bimonthly
	Van der Wekken *et al.* [38]	Case report	n = 1					0.032	0.088	0.36				C: mg/L
**PIs**														
Atazanavir/cobicistat														
	Momper *et al.* [18]	Non-randomized, open-label, parallel-group, multicenter prospective study	2T: n = 5<br>3T: n = 6	25.33 (20.95 – 27.32)	18.85 (11.90 – 31.48)	36.20 (24.09-46.14)	0.21 (0.16-0.28)	0.21 (0.11 – 0.56)	0.61 (0.42-1.03)	0.74 (0.53-1.04)	0.46 (0.19-1.11)	0.32 (0.20-0.51)	0.32 (0.11 – 0.94)	AUC: mg*h/L<br>C: mg/L<br>GMR (90% CI)
Darunavir/ritonavir (monotherapy)														
	Mandelbrot *et al.* [14]	One-arm, open-label, multicentre; Phase II clinical trial	T2: n = 80<br>T3: n = 73<br>At birth: n = 61				Total: 2250 (1414 - 3132)<br>Unbound: 182 (124 - 261)	Total: 2332 (1604-3107)<br>Unbound: 174 (123-265)						AUC: ng*h/mL<br>C: ng/mL<br>Dose: 600/100mg bid

Drug (group)	Reference	Design	Population	AUC			C_{trough}			GMR				Comments
				Second trimester	Third trimester	Postpartum	Second trimester	Third trimester	Postpartum	AUC		C_{trough}		
										2T/PP	3T/PP	2T/PP	3T/PP	
**INSTIs**														
Bictegravir														
	Zhang *et al.* [11••]	Open-label, single-arm, prospective phase 1b study	T2: n = 21<br>T3: n = 30<br>PP: n = 31	Total: 62.8 (32.2%)<br><br>Unbound: 0.224 (42.0%)	Total: 60.2 (29.1%)<br><br>Unbound: 0.219 (33.9%)	Total: 135 (26.9%)<br><br>Unbound: 0.354 (34.2%)	1.05 (45.2%)	1.07 (41.7%)	3.53 (38.4%)	Total, 0.45 (0.40-0.50)<br><br>Unbound, 0.62 (0.55-0.69)	Total, 0.44 (0.40-0.49)<br><br>Unbound, 0.62 (0.56-0.70)	0.27 (0.22-0.33)	0.30 (0.27-0.34)	$AUC_{tau(unbound)}$: (h*mg/ml) mean (%CV)<br>C_{trough}: mg/mL<br>GMR (90% CI)<br><br>Only samples of PP week 6 included.
	Van der Wekken *et al.* [21]	Multicenter, open-label, non-randomized trial	T3: n = 6<br>PP: n = 5		51.0 (21%)			1.0 (34%)			0.56 (0.41-0.77)		0.38 (0.29-0.49)	AUC_{tau}: (h*mg/ml) [GM (%CV)]<br>C_{trough}: mg/L<br>GMR (90% CI)
	Powis *et al.* [22]	Ongoing, non-randomized, open-label, parallel-group, multicenter, phase IV prospective study	n = 27	53.4 (49.4-70.3)	51.8 (38.8-66.9)	126 (80.3-140.0)	1.13 (0.59-1.37)	1.01 (0.69-1.21)	3.30 (1.81-3.78)	0.51 (0.37-0.70)	0.44 (0.37-0.53)	0.28 (0.19-0.40)	0.29 (0.23-0.37)	AUC_{tau}: (h*mg/ml)<br>C_{trough}: mg/L<br>GMR (90% CI)
Cabotegravir														
	Atoyebi *et al.* [29••]	In silico; full-body adult PBPK model	n/a	Dose 1: 944 (0.12)<br><br>Dose 2: 1726 (0.07)	Dose 1: 871 (0.13)<br><br>Dose 2: 1532 (0.08)	Dose 1: 1632 (0.07)<br><br>Dose 2: 2857 (0.04)	Dose 1: 1.27 (91.4)<br><br>Dose 2: 1.00 (116)	Dose 1: 1.21 (95.9)<br><br>Dose 2: 0.94 (123)	Dose 1: 2.20 (53.7)<br><br>Dose 2: 1.70 (68.9)	Dose 1: 0.58<br><br>Dose 2: 0.60	Dose 1: 0.53<br><br>Dose 2: 0.54	Dose 1: 0.58<br><br>Dose 2: 0.59	Dose 1: 0.55<br><br>Dose 2: 0.55	AUC: mg*h/L (CV%)<br>C: mg/L (CV%)<br><br>Dose 1: 600mg then 400mg monthly<br>Dose 2: 600mg bimonthly
	Van der Wekken *et al.* [28•]	Case report	n = 1					2.45	2.67	0.92				C: mg/L

Drug (group)	Reference	Design	Population	AUC			C_{trough}			GMR				Comments
				Second trimester	Third trimester	Postpartum	Second trimester	Third trimester	Postpartum	AUC 2T/PP	3T/PP	C_{trough} 2T/PP	3T/PP	
Dolutegravir														
	Momper *et al.* [24]	Open-label, opportunistic, parallel-group, multicenter, phase IV PK, and safety study	T2: n = 15<br>T3: n = 27<br>PP: n = 23				Unbound C_0: 6.3 (4.7-18.4)<br><br>Unbound%: 0.85 (0.76-0.98)	Unbound C_0: 8.0 (5.6-16.9)<br><br>Unbound%: 1.02 (0.78-1.14)	Unbound C_0: 13.3 (8.4-22.7)<br><br>Unbound%: 0.69 (0.49-0.88)			Unbound C_0: 0.39<br><br>Unbound %: 1.41	Unbound C_0: 0.77<br><br>Unbound %: 1.49	AUC: ng*h/mL<br>C: ng/mL<br><br>LLOQ = 1.56 ng/mL<br><br>4 samples below LLOQ
Raltegravir														
	Moreira *et al.* [25•]	Opportunistic study design	3T: n = 15<br>PP: n = 8		RAL: 6.29 (4.69–8.42)<br><br>RAL GLU: 5.46 (3.49–8.55)	RAL: 7.41 (4.49–12.21)<br><br>RAL GLU: 6.07 (3.29–11.18)		RAL: 50.12 (37.13–67.64)<br><br>RAL GLU: 65.87 (43.32–100.18)	RAL: 53.53 (36.52–78.45)<br><br>RAL GLU: 67.33 (24.54–184.74)		RAL: 0.85<br><br>RAL GLU: 0.90		RAL: 0.94<br><br>RAL GLU: 0.98	AUC: mg*h/L<br>C: ng/mL
	Carvalho *et al.* [26]	Prospective study	T2: n = 20<br>T3: n = 22				0.09	0.07					0.75	C: mg/L
**Pharmacoenhancers**														
Cobicistat														
	Momper *et al.* [36]	Non-randomized, open-label, parallel-group, multicenter, prospective study	2T: n = 5<br>3T: n = 7	7.39 (5.39-8.31)	4.89 (2.98-6.89)	9.38 (8.57-10.28)	6.3 (2.5-10.9)	6.2 (2.5-29.8)	28.6 (23.7-37.0)	0.65 (0.49-0.86)	0.48 (0.30-0.77)	0.17 (0.086-0.35)	0.46 (0.075-2.79)	AUC: mg*h/L (CV%)<br>C: mg/L (CV%)<br>GMR (90% CI)

AUC: area under the curve; Ctrough/C: trough concentration; GMR: geometric mean ratio; 2T: second trimester; 3T: third trimester; PP: postpartum; NRTI: nucleoside reverse transcriptase; n: number; AUCtau: AUC for dosing interval; CV%: coefficient of variation; CI: confidence interval; PK: pharmacokinetic; n/a: not applicable; Total: total concentration; Unbound: unbound concentration; Clast: last concentration; NNRTI: non-nucleoside reverse transcriptase inhibitor; LLOQ: lower limit of quantification; PBPK: physiologically based pharmacokinetic; PI: protease inhibitor; bid: twice daily; INSTI: integrase strand transfer inhibitor; GM: geometric mean; Unbound%: percentage of unbound drug; RAL: raltegravir; RAL GLU: raltegravir glucuronide; TAF: Tenofovir Alafenamide
